# Oscillatory dynamics in a model of vascular tumour growth - implications for chemotherapy

**DOI:** 10.1186/1745-6150-5-27

**Published:** 2010-04-20

**Authors:** IJ Stamper, MR Owen, PK Maini, HM Byrne

**Affiliations:** 1 Centre for Mathematical Medicine and Biology, School of Mathematical Sciences, University of Nottingham, Nottingham NG7 2RD, UK; 2 Centre for Mathematical Biology, Mathematical Institute, University of Oxford, 24-29 St Giles', Oxford OX1 3LB, UK; 3 Oxford Centre for Integrative Systems Biology, Dept of Biochemistry, South Parks Road, Oxford OX1 3QU, UK

## Abstract

**Background:**

Investigations of solid tumours suggest that vessel occlusion may occur when increased pressure from the tumour mass is exerted on the vessel walls. Since immature vessels are frequently found in tumours and may be particularly sensitive, such occlusion may impair tumour blood flow and have a negative impact on therapeutic outcome. In order to study the effects that occlusion may have on tumour growth patterns and therapeutic response, in this paper we develop and investigate a continuum model of vascular tumour growth.

**Results:**

By analysing a spatially uniform submodel, we identify regions of parameter space in which the combination of tumour cell proliferation and vessel occlusion give rise to sustained temporal oscillations in the tumour cell population and in the vessel density. Alternatively, if the vessels are assumed to be less prone to collapse, stable steady state solutions are observed. When spatial effects are considered, the pattern of tumour invasion depends on the dynamics of the spatially uniform submodel. If the submodel predicts a stable steady state, then steady travelling waves are observed in the full model, and the system evolves to the same stable steady state behind the invading front. When the submodel yields oscillatory behaviour, the full model produces periodic travelling waves. The stability of the waves (which can be predicted by approximating the system as one of *λ*-*ω *type) dictates whether the waves develop into regular or irregular spatio-temporal oscillations. Simulations of chemotherapy reveal that treatment outcome depends crucially on the underlying tumour growth dynamics. In particular, if the dynamics are oscillatory, then therapeutic efficacy is difficult to assess since the fluctuations in the size of the tumour cell population are enhanced, compared to untreated controls.

**Conclusions:**

We have developed a mathematical model of vascular tumour growth formulated as a system of partial differential equations (PDEs). Employing a combination of numerical and analytical techniques, we demonstrate how the spatio-temporal dynamics of the untreated tumour may influence its response to chemotherapy.

**Reviewers:**

This manuscript was reviewed by Professor Zvia Agur and Professor Marek Kimmel.

## Background

Experimental observations suggest that blood vessels in solid tumours may be affected by the mechanical stress exerted on them by tumour cells. Immature vessels lacking pericytes are especially likely to be structurally unstable [1]. As a tumour grows, the burden from tumour cells may cause the diameters of immature vessels in the tumour's interior to decrease [1,2]. Indeed, completely collapsed, or occluded, vessels - those with a closed lumen - are found more frequently at higher tumour cell densities [1,2]. On the other hand, if tumour cells are killed with cytotoxic therapy, the pressure on the vessels reduces, and vessel recovery may occur: the diameter of the vessels increases and the number of occluded vessels decreases [1,2].

The model we develop in this paper is of reaction-diffusion type and describes a vascularised tumour growing in a one-dimensional spatial domain. We use the model to investigate how tumour cells and blood vessels may interact and influence the overall tumour growth dynamics. We assume that the tumour relies on oxygen delivered via the vasculature for sustained growth. The vascular density increases due to tumour angiogenesis, while it decreases due to vessel destabilisation and occlusion at a rate which is an increasing function of the ratio of tumour cell density to vessel surface area (reflecting the experimental observations mentioned above). Previously, other authors have used mathematical models to study vessel occlusion in tumours. As in our model, in the multiphase model developed by Breward *et al. *[3] angiogenesis and vessel occlusion act as opposing forces that regulate vessel growth, with vessel occlusion being switched on smoothly when the pressure exerted on the vessels by the cells exceeds a critical value. Although their model couples vessel and tumour cell dynamics, no oscillatory behaviour was reported - only stable growth dynamics were observed [3]. An alternative approach to modelling vessel occlusion was proposed by Araujo and McElwain, who developed a continuum-mechanical model in which vascular collapse is linked to the evolution of residual stress within the growing tumour [4]. In this model interactions between the tumour and (the implicit) vasculature (which is represented by a prescribed nutrient distribution) may generate oscillations in the tumour radius, albeit of very small amplitude [4].

Analysis of the model we derive in this paper reveals that the interplay between a tumour and its blood vessels may lead to strongly oscillatory dynamics. In particular, oscillations in the model variables arise if the vessels are structurally unstable, and thus are prone to occlusion (as opposed to stiffer and less compliant vessels which yield stable steady state solutions). The oscillations in our model are sustained by repeated cycles of tumour expansion and vessel occlusion, followed by episodes of tumour cell death and vessel recovery. As the tumour cells spread and colonise the spatial domain, regular or irregular spatio-temporal oscillations develop. These complex spatio-temporal dynamics may contribute to the reported spatio-temporal heterogeneity that characterises many vascularised solid tumours. Previously, oscillatory dynamics have been reported, for example by Arakelyan *et al.*, who developed a spatially-averaged ordinary differential equation (ODE) model of tumour growth [5]. Validation of the model was obtained from MRI data describing the growth of implanted human ovarian carcinoma [6]. Furthermore, spatio-temporal irregularities have been observed in models which describe various aspects of the immune response to tumours [7-9].

Although our model does not explicitly account for blood flow, the highly irregular oscillations in vascular density and oxygen levels that the model generates are consistent with oxygen concentrations in tumours whose blood flow is chaotic. Chaotic blood flow is believed to be one of the main barriers to drug delivery [10], and the decreased vascular surface area and impaired blood flow of occluded vessels may also be detrimental to delivery [2,11]. So as to gain insight into therapeutic implications, we investigate the impact of chemotherapy on the tumour. We focus on the cytotoxic drug doxorubicin, a widely used anti-cancer agent which may be used in free form or encapsulated in liposomes [12,13]. Several models of doxorubicin administration have been developed [12,14,15]. For example, in [12] a compartmental model is used to compare the efficacy of free doxorubicin delivery versus liposome-bound delivery. The model in [12] distinguishes between vascular (plasma), extracellular and intracellular doxorubicin concentrations, accounting for the fact that the drug must enter the cells to be effective. A similar approach is used in [14] where cell kill depends on the amount of doxorubicin trapped within the cells. However, neither model accounts for both tumour cell proliferation and vascular remodelling, thus neglecting any effects that interactions between these phenomena are likely to induce.

On the contrary, our investigation concentrates on applying chemotherapy in different tumour growth scenarios, and shows how the intrinsic system dynamics (oscillatory or non-oscillatory, corresponding to compliant or non-compliant vessels, respectively) may alter treatment outcome. Specifically the ways in which the system recovers from therapy are found to vary markedly. This is consistent with simulations in [15] where the dynamics of tumour growth were modelled using a multiscale hybrid cellular automaton, and where the system's therapeutic response was influenced by the vessels' maturation status.

We organise this paper as follows. In Methods we develop our model of vascular tumour growth. In Results we show how, in a spatially homogeneous version of our model (in which spatial effects are neglected and fast kinetics are assumed for oxygen), the tumour cell population and the vessel density evolve to a stable co-existence equilibrium, or, if the steady state is unstable, undergo oscillations (the latter occurring when the maximum rate of vessel occlusion exceeds a critical value). Then, by reintroducing spatial effects, we investigate the way in which a small population of tumour cells seeded at one end of the domain invades the tissue. In agreement with analysis of other reaction-diffusion systems, we find that the form of the solutions behind the invading tumour cell front depends on the stability of the spatially homogeneous co-existence steady state behind the front [16]. Specifically, when the co-existence steady state is stable, the system evolves to this stable equilibrium in the wake of the invading front. In contrast, in the case of an unstable co-existence steady state, the system is subject to either regular or irregular oscillations. We are able to predict the type of oscillations that will arise for particular model parameters by analysing a system of *λ*-*ω *type that approximates the dynamics close to the unstable steady state; this approach has previously been applied by Sherratt [17]. We then investigate the effect of chemotherapy. We conclude with a discussion of our results, and suggestions for future research.

## Methods

The model we develop describes the growth of a solid tumour embedded in a vascularised tissue in a one-dimensional spatial domain of length *L*; this can be thought of as a two-dimensional domain which is spatially averaged in one direction. The spatial variable is denoted by *x*, so that 0 ≤ *x *≤ *L*. We assume that the tissue composition can be described by the following dependent variables: the density of tumour cells, *p*(*x, t*); the vascular density, *v*(*x, t*); and the concentration of oxygen, *s*(*x, t*). The units of *p *are taken to be cells per unit volume. Following [18] the vascular density is measured in units of vascular surface area per unit volume. Furthermore, following [19], we measure the oxygen concentration in units of volume of oxygen per unit volume. To model chemotherapy we introduce the variables *c*_*b*_(*t*) and *c*(*x, t*) which denote the concentration of drug in the bloodstream and in the tumour tissue, respectively. The units of these concentrations are taken to be mass per unit volume. While *c*_*b*_(*t*) is prescribed and depends on the delivery mechanism being used (single bolus or multiple boluses), the evolution of the other model variables *u *(*u *= *c, p, v, s*) is determined by applying the principle of mass balance which gives that each *u *satisfies a partial differential equation (PDE) of the following form (see e.g. [20]):(1)

where *J*_*u *_denotes the flux and *f*_*u *_represents the net sources and sinks of species *u*. In what follows we adapt equation (1) for our dependent variables.

### Tumour cells, *p*(*x*, *t*)

For tumour cells, the function *f*_*p *_incorporates cell division and cell death. Oxygen is assumed to be the single rate-limiting nutrient that regulates the rate of tumour cell proliferation. Since we expect that the rate at which the tumour population increases by cell division is a saturating function of oxygen [21], we assume that the proliferation rate is of Michaelis-Menten type (see e.g. [20]), given by

where *β *is the maximum proliferation rate per unit of cell density and *s*_*β *_is the level of oxygen at which the proliferation rate is half-maximal. We assume that cell death occurs naturally due to apoptosis at rate *d*_*p*_*p*, where *d*_*p *_is a positive constant. To account for tumour cell death induced by the cytotoxic drug, we assume that the drug kills proliferating cells in a concentration-dependent manner [22] and that the maximum rate of cell kill is equal to the rate of proliferation (a similar assumption for the action of a cytotoxic drug was used in [23]). We note that *c *= 0 implies that the rate of cell proliferation is unaffected (i.e. all cells are proliferating and there is no drug-induced cell kill), *c *= *K*_*c *_implies zero net contribution from proliferation (i.e. proliferation has ceased in half the cell population, which instead dies), and *c *→ ∞ implies no contribution from proliferation (i.e proliferation has ceased in all cells, which instead die). The precise mechanism by which doxorubicin is anti-proliferative is not known, but it is in part due to intercalation into DNA [22].

Regarding tumour cell movement, we allow for random motion of the cells and so specify , where *D*_*p *_is the random motility co-efficient which is assumed to be constant.

Taking these factors into account, we deduce that the density of tumour cells, *p*(*x, t*), evolves according to the following PDE:(2)

where *K*_*c *_is a positive constant denoting the concentration at which the cell kill is half-maximal and *D*_*p*_, *β*, *s*_*β *_and *d*_*p *_are positive constants.

### Tumour vasculature, *v*(*x*, *t*)

While not explicitly introducing VEGF or any other angiogenic factor into our model, we assume that the tumour stimulates angiogenesis in such a way that the vasculature undergoes logistic growth characterised by a constant rate of proliferation, *η*_0_, and a carrying capacity, *V*_0_. We note here that a more realistic model of tumour-induced angiogenesis could include VEGF (e.g. VEGF could be secreted by the tumour cells, especially when the level of oxygen is low). The level of angiogenesis could then be linked explicitly to the concentration of VEGF, for example by allowing *η*_0 _to be an increasing, saturating function of VEGF. However, since our goal is to gain insight into the dynamics that arise from tumour cell proliferation and vessel occlusion, we refrain from adding this extra complexity and assume that *η*_0 _is constant (equivalent to VEGF being maintained at a constant level).

Following [1], where occluded vessels were typically surrounded by more tumour cells than open vessels, we assume that the tumour vessels may become occluded if the pressure exerted on them by the tumour cells becomes too large. In particular, we take the rate of occlusion to be a saturating function of Michaelis-Menten type of *p/v*, the ratio of tumour cells per vessel surface area so that the rate of vessel occlusion is given by

where the constant *K*_*δ *_denotes the value of *p*/*v *at which the rate of occlusion attains its half-maximal value, *δ*/2.

To account for movement of the endothelial cells (ECs) that constitute the vessels we include random motion, assuming that this occurs with a constant diffusivity co-efficient, *D*_*v*_. Since VEGF is not explicitly included in the model we neglect EC movement due to chemotaxis.

Assembling the above terms gives the following PDE for the density of tumour vasculature, *v*(*x, t*):(3)

where *D*_*v*_, *δ*, *K*_*δ*_, *η*_0 _and *V*_0 _are positive constants.

### Oxygen level, *s*(*x*, *t*)

We assume that the concentration of oxygen, *s*(*x, t*), increases due to delivery from the vasculature. Transcapillary exchange of molecules from the blood occurs mainly by diffusion and convection [24]. For simplicity we suppose that diffusive effects dominate and that the diffusive flux across the vessel wall is proportional to the vascular surface area and to the difference between the concentrations of the substance in the plasma and in the interstitium [24]. Thus we model the delivery of oxygen from the blood as *h*_*s*_(*s*_*b *_- *s*)*v*, where *h*_*s *_is the vascular permeability constant [24], *s*_*b *_is the (assumed constant) concentration of oxygen in the blood, *s *is the concentration of oxygen in the tissue and *v *is the vascular surface area per unit tissue volume as explained above. We note that as the oxygen flows through the tissue the level of oxygen in the blood, *s*_*b*_, decreases along each vessel due to the diffusive flux into the tissue. However, since we do not account for vessel morphology we make the simplifying assumption that *s*_*b *_is a constant.

After transcapillary transfer, the transport of oxygen within the interstitium occurs by diffusion and convection [24]. Neglecting the convective part for simplicity, we assume that the transport of oxygen within the tissue is via diffusion only so that .

We assume that oxygen is consumed by tumour cells, at rate *σ*_*p*_*ps*, and by other cellular species (e.g. fibroblasts and macrophages) resident in the tissue, at rate *d*_*s*_*s*. An alternative form for tumour oxygen consumption could be obtained by assuming that the consumption rate is of Michaelis-Menten type. By assuming that *d*_*s *_> 0 we ensure that, in the absence of tumour cells, the level of oxygen evolves to a finite, tissue-specific equilibrium value. The particular value of the parameter *d*_*s*_, and the associated equilibrium oxygen value, will be tissue-specific and could vary as the tumour and the surrounding stroma evolve.

When the above factors are combined, the PDE describing the tissue oxygen concentration, *s*(*x, t*), is given by:(4)

where *D*_*s*_, *h*_*s*_, *s*_*b*_, *σ*_*p *_and *d*_*s *_are positive constants.

### Plasma level of drug, *c*_*b*_(*t*)

We assume that chemotherapy is administered into the bloodstream either as a single bolus or as repeated doses. We assume that only free doxorubicin is being administered and that its concentration in the plasma decays exponentially, so-called one-compartment modelling [15]. We denote by *c*_*b*_(*t*) the prescribed concentration of drug in the bloodstream and write(5)

in the case of a single bolus and(6)

in the case of multiple injections of period *T*_*per*_. In (5) and (6), *c*_*init *_is the initial value of the blood drug concentration after a single bolus. Furthermore, the constant *k *is related to the drug's half-life in blood, *T*_1/2_, via

so that the drug decays exponentially after being injected. In the case of multiple injections we assume that the doses are such that the plasma concentration regains its initial value at each injection (achieved mathematically in (6) by making the time variable modulo *T*_*per*_). More precisely, we assume *T*_1/2 _≪ *T*_*per*_, as is the case for doxorubicin administration, where the half-life is roughly one day and the period is on the order of weeks [15].

### Concentration of drug in tumour tissue, *c*(*x*, *t*)

Guided by equation (4), we assume that *c*(*x, t*), the concentration of drug in the tumour, evolves according to the following equation:(7)

where *D*_*c*_, *h*_*c *_and *d*_*c *_are positive constants. In equation (7) the vascular delivery term is similar to that introduced for oxygen in (4), while the decay term represents drug uptake by both tumour cells and other cells present in the extracellular matrix.

### Boundary and initial conditions

To summarise, our model comprises four coupled PDEs defined by equations (2)-(4) and (7) and prescribed plasma drug concentrations given by either equation (5) or (6). For simplicity, and in order to understand the dynamics, we consider a closed or isolated tissue in which all behaviour arises due to the interaction terms. Thus we impose no-flux boundary conditions of the form:(8)

where *T*_*fin *_> 0 is an arbitrary time. These boundary conditions imply that no cells, vasculature, oxygen or drug leave the system through the tissue boundaries. Unless stated otherwise, we use the following initial conditions:(9)

where *p*_*i *_is an arbitrary constant and *s*_*i *_= *h*_*s*_*s*_*b*_*v/*(*h*_*s*_*v *+ *σ*_*p*_*p *+ *d*_*s*_). This implies that the tumour cells are introduced at the left-hand boundary of a tissue in which the vasculature is at its carrying capacity, *V*_0_, and the oxygen level is at quasi-steady state. In addition there is no drug in the tissue initially. Before analysing the model it is convenient to nondimensionalise it as explained in the next section.

### Nondimensionalisation

To nondimensionalise equations (2)-(6) and (7) we introduce the following dimensionless variables:(10)

Using the above rescalings the model equations become:(11)

where

are dimensionless parameters. A discussion of the parameter values that we use is contained in section A of the Appendix (additional file 1).

## Results

We first investigate the behaviour of a spatially homogeneous submodel. We then reintroduce tumour cell and EC movement and use *λ *- *ω *analysis to obtain insight into the spatio-temporal dynamics. We finish by considering the response of the full system to chemotherapy.

### Spatially homogeneous submodel - no therapy

We obtain our spatially homogeneous submodel by assuming that no chemotherapy is applied and by neglecting spatial effects, i.e. assuming that *c *= *c*_*b *_= 0 and that the variables in (12)-(14) are functions only of time. We also assume that the timescales inherent in the oxygen equation are short in comparison to the timescale for tumour growth (as for instance assumed in [15]), i.e.(16)

Under this assumption (14) is in a quasi-steady state so(17)

where(18)

are dimensionless parameter groupings. In what follows we omit the asterisks and tildes in (12)-(14) and (17) for notational convenience. After substituting (17) into (12), our model reduces to the following pair of ODEs for *p *and *v*:(19)

which can be analysed using phase plane techniques. By setting the derivative in (19) equal to zero we deduce that the *p*-nullclines are:(21)

and(22)

We note that *f *(*v*) is a straight line. From (19) it follows that ∀*p *> 0,  < 0 if *p *>*f *(*v*) and  > 0 if *p *<*f *(*v*). Therefore, if 1 - *d*_*p*_*s*_*β *_- *d*_*p *_≤ 0, then ∀*v *> 0, *f *(*v*) < 0, and no physically realistic co-existence steady state exists. Instead the tumour decreases in size ∀*v *> 0 (since  < 0), and the system evolves to the linearly stable tumour-free steady-state solution, (*p, v*) = (0, 1). So that a physically realistic co-existence steady state may exist, we require *d*_*p *_< 1/(1 + *s*_*β*_), meaning that the apoptotic rate of the tumour cells is less than the maximum rate of proliferation.

Similarly, setting (20) equal to zero we deduce that the nontrivial *v*-nullcline is:(23)

**Figure 1:**
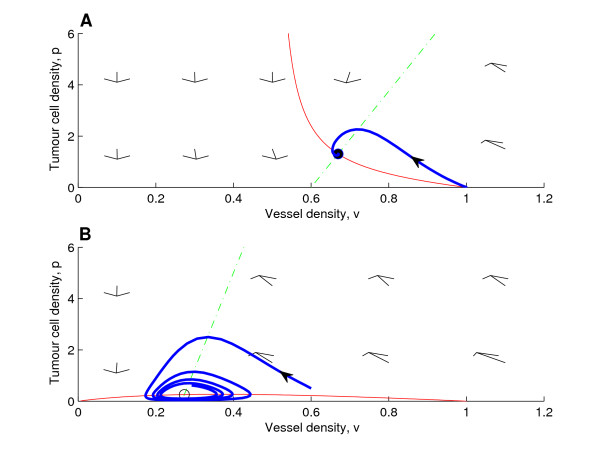
**Typical phase planes**. **(A) **Typical phase plane with (non-zero) nullclines for case (a), here for *δ*/*η*_0 _< 1, showing a steady state which is a stable spiral. **(B) **Typical phase plane with (non-zero) nullclines for case (b), *δ*/*η*_0 _> 1. The steady state is unstable and the shown trajectory evolves into a stable limit cycle. We remark that compared to **(A) **we have varied *δ*, *d*_*s *_and *σ*_*p *_(*d*_*s *_and *σ*_*p *_were chosen so that the co-existence steady state would be unstable). **Key**: green dashed-dotted lines: non-zero *p*-nullclines; solid red lines: non-zero *v*-nullclines; open circle: linearly unstable steady state; closed circle: linearly stable steady state; blue bold lines with solid arrow: trajectories for the initial conditions (*p*(0), *v*(0)) = (0.01, 1) (**A**) and (*p*(0), *v*(0)) = (0.5, 0.6) (**B**). **Parameter values**: *η*_0 _= 0.02, *d*_*p *_= 0.8, *d*_*s *_= 0.9, *δ *= 0.01, *s*_*β *_= 0.1 and *σ*_*p *_= 0.08 (**A**) and *η*_0 _= 0.02, *d*_*p *_= 0.8, *d*_*s *_= 0.4, *δ *= 0.03, *s*_*β *_= 0.1 and *σ*_*p *_= 0.01 (**B**).

It is straightforward to show that *g*(*v*) is positive only if 0 ≤ *v *≤ 1 (see Figure 1 where the *v*-nullclines are indicated by solid red lines). Therefore, for the *p*- and *v*-nullclines to intersect and a co-existence steady state to exist, the zero of *f *(*v*), *f*_*zero*_, must fulfill(24)

as follows from (22) (if *f*_*zero *_≥ 1, the system evolves to the linearly stable tumour-free steady state (*p, v*) = (0, 1)). For smaller values of *f*_*zero *_(0 <*f*_*zero *_< 1), the stability of the co-existence steady state depends on the ratio *δ*/*η*_0 _in (23).

**Case (a)**: *δ*/*η*_0 _≤ 1

If *δ*/*η*_0 _= 1, then *g*(*v*) is a straight line, *g*(*v*) = 1 - *v*. As shown in Figure 1 (panel A) if *δ*/*η*_0 _< 1, then *g*(*v*) is a monotonically decreasing function for *v *> 1 - *δ*/*η*_0_, reaching zero at *v *= 1 (with *g*(*v*) < 0 for *v *< 1 - *δ*/*η*_0_). Since  > 0 for *p *<*g*(*v*) and  < 0 for *p *<*g*(*v*) (as follows from (20)), we have that at the steady state *V*_*p *_< 0 and *V*_*v *_< 0, the subscripts denoting partial derivatives and the function *V *given by (20). Because at the steady state the *p*-nullcline is such that *P*_*p *_< 0 <*P*_*v*_, it follows that *tr*(*A*) = *P*_*p *_+ *V*_*v *_< 0 and *det*(*A*) = *P*_*p*_*V*_*v *_- *P*_*v*_*V*_*p *_> 0, where *A *= (*a*_*ij*_) is the Jacobian matrix, implying that the co-existence steady state is always linearly stable. In Figure 1 (panel A) for a case where the steady state is a stable spiral, we show the phase plane, the nullclines and a trajectory with initial conditions corresponding to the introduction of a small number of tumour cells into a tissue with vasculature at carrying capacity ((*p*(0), *v*(0)) = (0.01, 1)). The phase planes were calculated numerically using MATLAB, and equations (19)-(20) were integrated using a Runge-Kutta method.

**Case (b)**: *δ*/*η*_0 _> 1

**Figure 2:**
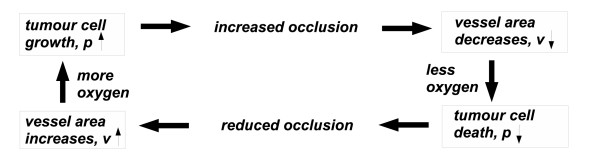
**Illustration of the mechanims causing oscillatory solutions**. Diagram explaining how tumour cell proliferation and vessel occlusion sustain the limit cycle oscillations in system (19)-(20). The expanding tumour cell population occludes vessels, leading to reduced oxygen levels and reduced tumour cell division. The shrinking tumour cell population allows vessel recovery and the cycle repeats.

The function *g*(*v*) is concave for *v *≥ 0 with *g*(0) = 0 and *g*(1) = 0 (see Figure 1, panel B). In this case oscillatory solutions are possible. In Figure 1 we present a typical phase plane for a case where the steady state is an unstable spiral and a stable limit cycle exists. The mechanism responsible for the oscillations is vessel occlusion by the tumour cells. As the tumour cells proliferate and increase in number, more vessels become occluded. Occlusion reduces the level of oxygen and hence the rate of tumour cell proliferation which results in fewer tumour cells. The reduction in tumour burden allows the vessels to recover. Vessel recovery leads to increased oxygenation and, therefore, an increase in the rate of tumour cell proliferation and the size of the tumour cell population. With the expanding tumour mass, occlusion once again becomes important, causing the cycle to repeat (see Figure 2 for a schematic explanation). In fact, the oscillations in this submodel are similar to those observed in "predator-prey" systems (see e.g. [20]); here the tumour cells act as "predators" by occluding vessels, "the prey".

We remark that it is the particular functional form of  ≡ *V*, which appears in (20), that gives rise to oscillatory solutions; other choices of  ≡ *V *may not admit oscillations. In particular, we require *V*_*v *_> 0 so that an unstable steady state with a limit cycle may exist. According to the Hopf bifurcation theorem, an unstable steady state with a stable limit cycle, and thus oscillations, may appear as the bifurcation parameter is varied so that the real part of a pair of complex conjugate eigenvalues of the Jacobian matrix changes sign [20]. This corresponds to a co-existence steady state changing from a stable spiral to an unstable spiral (or vice versa). Since *trA *= *P*_*p *_+ *V*_*v *_> 0 is a necessary condition for an unstable spiral and *P*_*p *_< 0, we need *V*_*v *_> 0 for the existence of an unstable spiral, implying that it can only exist where *g*(*v*) has positive slope (for the problem at hand, where the slope of *g*(*v*) is positive,  < 0 to the left of the nullcline and  > 0 to the right; thus *V*_*v *_> 0). In Figure 1, if the *p*-nullcline is moved to the right, causing the zero of *f *(*v*), *f*_*zero*_, to increase, the co-existence steady state changes stability, and the stable limit cycle disappears via a Hopf bifurcation. It is also possible to obtain a Hopf bifurcation by increasing the rate of oxygen consumption by tumour cells, *σ*_*p*_, thus decreasing the slope of the *p*-nullcline. We remark that the oscillatory dynamics close to the Hopf bifurcation can be approximated by the corresponding normal form of *λ*-*ω *type (see below, and section B in the Appendix (additional file 1)).

To summarise, in this section we have shown how the existence and stability of the co-existence steady state depend on the model parameters. In particular, oscillatory solutions may occur when *δ*, the maximum rate of occlusion, is sufficiently high; if *δ *exceeds the maximum rate of endothelial cell proliferation, *η*_0_, then the co-existence steady state may be unstable and periodic oscillations are possible. We remark that a high value of *δ *corresponds to immature vessels that are more prone to collapse.

### Spatial model with random motion of tumour cells and endothelial cells - no therapy

In this section we re-introduce spatial effects (deferring the inclusion of oxygen diffusion until we investigate chemotherapy). We continue to assume that oxygen is at quasi-steady state and does not diffuse, so that *s *is defined in terms of *v *and *p *by (17) and equations (12)-(13) become:(25)

In what follows we suppose that a small mass of tumour cells is introduced at one side (*x *= 0) of a homogeneous vascularised tissue which is otherwise devoid of tumour cells ((*p*(*x*, 0), *v*(*x*, 0)) = (0, 1) ∀*x *≠ 0). This situation could describe (i) tumour cells implanted in an experiment, (ii) metastatic cells that have entered the tissue from the supporting vasculature, or (iii) cells in an avascular tumour that arose spontaneously within a tissue. Due to random movement, the tumour cells may spread to and colonise parts of the tissue that were initially unaffected. We analyse cases for which the co-existence steady state must be linearly stable (case (a) above, corresponding to *δ*/*η*_0 _≤ 1), before studying cases for which it may be unstable (case (b) above, corresponding to *δ*/*η*_0 _> 1). In the case of an unstable steady state we note that when the random motion of endothelial cells is equal to that of tumour cells (*D*_*v *_= 1), the system lends itself to *λ*-*ω *analysis. For mathematical tractability, we carry out the *λ*-*ω *approximation for the simplified case in which the rate at which the tumour cells consume oxygen is negligible (*σ*_*p *_= 0 in (25)). Numerical simulations with *D*_*v *_≠ 1 and *σ*_*p *_≠ 0 reveal qualitatively similar dynamics.

To obtain numerical solutions to the system (25)-(26) we use the NAG routine D03PCF in Fortran [25]. This routine utilises finite differences for the spatial discretisation. Specifically, the method of lines is used to reduce the governing PDEs to a system of ODEs, which is then solved using a backward differentiation method. In each figure legend we state the mesh size Δ*x*.

#### Stable co-existence steady state

When the co-existence steady state is stable, diffusion driven instabilities (DDI) may occur for certain parameter values provided that the diffusion co-efficients differ (*D*_*v *_≠ 1) [26]. Analysis of our system shows that DDI can only occur if *D*_*v *_< 1 and we have used parameter values such that the system is driven unstable. However, for brevity, we do not present those simulations or analyse this case further.

We have carried out simulations of equations (25)-(26) for several cases in which the underlying co-existence steady state is linearly stable and the random motility co-efficients for tumour and endothelial cells are equal (*D*_*v *_= 1 and DDI is not possible). In general these simulations show that when a population of tumour cells is introduced at the left-hand boundary, it invades the domain and the system evolves to its stable steady state behind the invading front. At large times the domain becomes spatially-uniform and the system reaches a stable steady state.

#### Unstable co-existence steady state

We now consider the case for which the spatially-uniform (no diffusion) model possesses an unstable co-existence steady state, with predator-prey-like oscillatory solutions (and with the tumour cells acting as "predators"). In [17] Sherratt studied a predator-prey reaction-diffusion model which, like ours, admits an unstable co-existence steady state, with a stable limit cycle. His analysis focussed on what happens when a small number of predators is introduced into a spatially-homogeneous population of prey. Behind the invading front, which connects the stable predator-free equilibrium ahead of the wave with the unstable co-existence equilibrium, two types of oscillatory wakes were seen: regular spatio-temporal waves and highly irregular oscillations [17]. In general, the invading front decays exponentially, with respect to the spatial variable, to the co-existence equilibrium and, if the system is close to the Hopf bifurcation, it stays there for a significant time (even though the equilibrium is unstable) before developing into a periodic wave train [27]. The stability of the resulting wave train determines whether the oscillations remain periodic (in the case of a stable wave train) or become irregular (in the case of an unstable wave train) [17,27].

**Figure 3:**
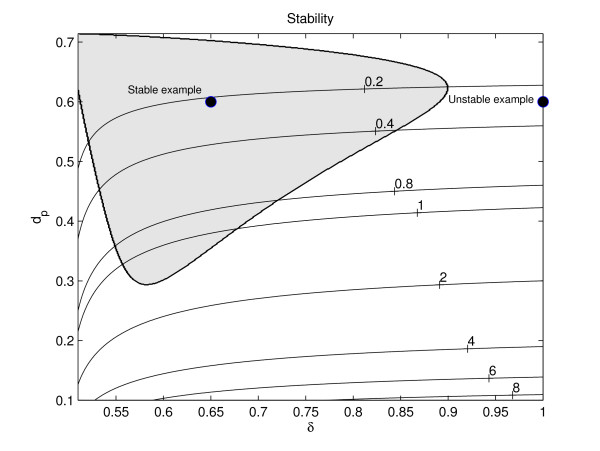
**Partition of (*δ*, *d*_*p*_)-space based on stability of wave trains**. Figure showing where in (*δ*, *d*_*p*_)-space (as determined by (A-7) in the Appendix (additional file 1)) the wake behind the invading wave of tumour cells is stable (shaded region) and where it is unstable (white region). The stability analysis requires *d*_*s *_≈ , where  denotes the value of *d*_*s *_at which the Hopf bifurcation occurs; thus in our (*δ*, *d*_*p*_)-space we mark by solid lines contours where  (as calculated from (A-11) in the Appendix (additional file 1)) is constant (corresponding values are labelled on the contours). For a case of stability ((*δ*, *d*_*p*_) = (0.65, 0.6) and *ds *≈ 0.2) tumour cell invasion into the spatially homogeneous vessel-only steady state results in regular spatio-temporal oscillations (see Figure 4), while in the case of instability ((*δ*, *d*_*p*_) = (1.0, 0.6) and *ds *≈ 0.2), irregular spatio-temporal oscillations develop (see Figure 5). **Key**: shaded region: wave stability; white region: wave instability; solid lines: lines where  is constant. **Parameter values**: *η*_0 _= 0.5, *s*_*β *_= 0.4 and *σ*_*p *_= 0.

Following [17,27] (see also [28]) we analyse equations (25)-(26) (here with *σ*_*p *_= 0 and *D*_*v *_= 1) by using normal form analysis to approximate it by one of *λ*-*ω *type near the Hopf bifurcation. The result of our analysis (for details of the analysis see section B in the Appendix (additional file 1)) is a prediction about the stability of the periodic waves behind the invading front. By keeping all other parameters fixed, and also ensuring that the system is close to Hopf bifurcation, it is possible to show that the stability of a wave train depends solely on the maximum rate of vessel occlusion, *δ*, and the tumour apoptosis rate, *d*_*p*_. In Figure 3, we have partitioned (*δ*, *d*_*p*_)-space into different regions according to whether the wave behind the invading front is stable (in the shaded region) or unstable (in the white region). We remark that once the other parameters have been fixed, the value of *d*_*s*_, the oxygen consumption rate of other cellular components, determines whether the system is near Hopf bifurcation, and thus whether the stability prediction is reliable.

**Figure 4:**
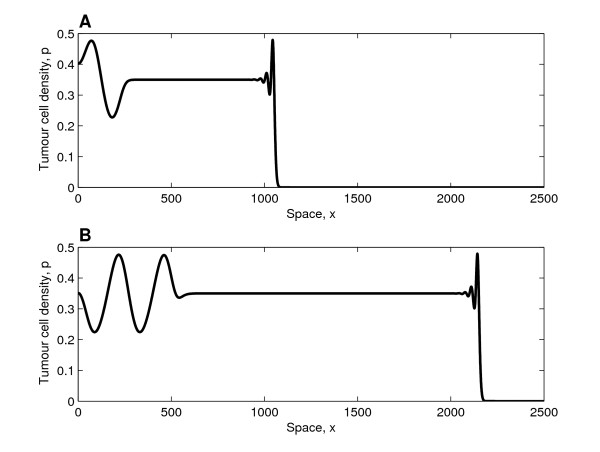
**Solution profiles in the case of stable wave trains**. Series of profiles (solutions of equations (25)-(26)) showing how the tumour cell density evolves when regular waves develop behind the invading tumour front (similar profiles of the vessel density not presented). Panel A (B) depicts the behaviour at *t *= 2000 (*t *= 4000). Behind the invading front, which travels with constant shape and connects the tumour-free steady state with the unstable co-existence steady state, regular spatio-temporal oscillations develop. Since the invading front moves faster than the evolving regular wave train, a large portion of the domain is at the unstable steady state ((*p, v*) = (0.35, 0.3)). **Parameter values**: *η*_0 _= 0.5, *d*_*p *_= 0.6, *d*_*s *_= 0.2,  = 0.2163 (using (A-11) in the Appendix (additional file 1)), *δ *= 0.65, *D*_*v *_= 1, *s*_*β *_= 0.4 and *σ*_*p *_= 0. For these parameter values the waves are stable (see Figure 3). For the numerical simulations we fix Δ*x *= 1/3 and *L *= 3000 with (*p*(0, 0), *v*(0, 0)) = (0.01, 1) and (*p*(*x*, 0), *v*(*x*, 0)) = (0, 1) for *x*: 0 <*x *≤ 1.

In Figure 4, we present a simulation for which the *λ*-*ω *analysis, as presented in Figure 3, predicts stable periodic travelling waves behind the invading front. We note that damped oscillations connect the tumour-free steady state ahead of the invading front with the co-existence steady state, a solution which is qualitatively similar to those presented in [17]. Since the co-existence steady state is unstable, oscillations develop; in this case these are regular spatio-temporal waves because the periodic wave is stable. By comparing panels A and B in Figure 4, we see that the portion of the spatial domain in which the system is at the co-existence steady state increases over time; thus the spatial domain appears to become transiently spatially uniform. As established for a predator-prey system in [29], this occurs because the speed of the interface between the "plateau" (where the system is at steady state) and the region of regular oscillations is less than the speed of the invading front. We remark that for large times, after the invading front has reached the right-hand boundary, regular temporal oscillations persist at fixed spatial positions. After the invading front has reached the right-hand boundary it is reflected (due to the no-flux boundary conditions), and regular waves, travelling in the opposite direction to that of the original front, develop. The no-flux conditions imply that the boundaries are impermeable to the tumour cells, an assumption whose validity depends on the tissue of interest. For example, while cancer cells may be able to colonise a softer tissue, a more rigid material, such as bone, may halt cancer invasion.

**Figure 5:**
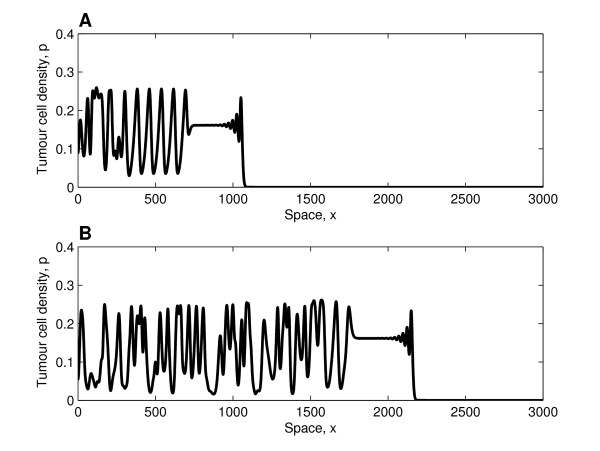
**Solution profiles in the case of unstable wave trains**. Series of profiles (solutions of equations (25)-(26)) showing how the tumour cell density evolves when irregular waves develop behind the invading tumour front (similar profiles of the vessel density not presented). Panel A (B) depicts the behaviour at *t *= 2000 (*t *= 4000). Behind the invading front, which travels with constant shape and connects the tumour-free steady state with the unstable co-existence steady state ((*p, v*) = (0.16, 0.3)), irregular spatio-temporal oscillations develop. **Parameter values**: *η*_0 _= 0.5, *d*_*p *_= 0.6, *d*_*s *_= 0.2,  = 0.2761 (using (A-11) in the Appendix (additional file 1)), *δ *= 1.0, *D*_*v *_= 1, *s*_*β *_= 0.4 and *σ*_*p *_= 0. For these parameter values the waves are unstable (see Figure 3). For the numerical simulations we fix Δ*x *= 1/3 and *L *= 3500, with (*p*(0, 0), *v*(0, 0)) = (0.01, 1) and (*p*(*x*, 0), *v*(*x*, 0)) = (0, 1) for *x*: 0 <*x *≤ 1.

In Figure 5 the waves that develop in the wake of the invading front are unstable, and irregular dynamics are observed. Since the system is close to the Hopf bifurcation, i.e. it is weakly unstable, the oscillations that develop behind the invading front are initially regular and only become irregular at later times (similar behaviour was observed in [17]). Once the cancer cells have colonised the entire tissue, irregular spatio-temporal oscillations persist.

In the above simulations oxygen diffusion was neglected and oxygen was assumed to be at quasi-steady state. The simulations were repeated for the full system, as defined by equations (12)-(14), with *c *= 0 in (12) and the following choice of parameter values: *D*_*v *_= 1,  and , wherein  and  correspond to the values of *d*_*s *_and *σ*_*p *_used in (25), as follows from (18). Guided by the discussion in section A of the Appendix (additional file 1), in our simulations of the full system we fixed *D*_*s *_= 2 × 10^5 ^and *h*_*s *_= 1.6 × 10^6^. The solutions of the full system for both stable and unstable co-existence steady states were qualitatively similar to those obtained for the simpler system (25)-(26) (and are not presented). We therefore conclude that the quasi-steady state assumption for oxygen is a good approximation, provided that the values of *D*_*s *_and *h*_*s *_are as derived in section A of the Appendix (additional file 1). Nevertheless, for completeness in the simulations of chemotherapy that follow we simulate the response of the full system (11)-(15).

### Full model - the effect of chemotherapy

Given that the maximum rate of occlusion, *δ*, determines the intrinsic dynamics of our system (once the other parameters have been fixed), below we investigate how vessel status (*δ *low or high) influences therapeutic response. We simulate the response of different tumours to chemotherapy, comparing cases for which the system dynamics behind the invading front prior to treatment result in (i) a stable equilibrium, (ii) regular waves, and (iii) irregular waves. The only parameter change that is varied between these three cases is *δ*.

**Figure 6:**
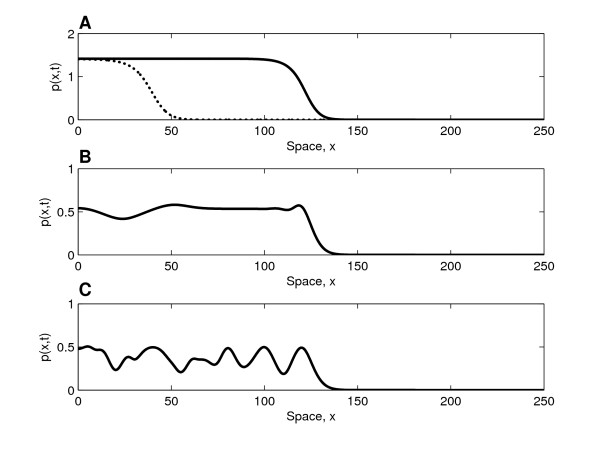
**Solution profiles prior to therapy**. Three snapshots of the spatial domain showing how the tumour cell density varies in space at the point in time (*t *= 200 in dimensionless units) at which chemotherapy is applied; the results of chemotherapy in these cases are presented in Figure 7. **(A) **Solid line depicts the cell density at the point in time at which chemotherapy is applied (*t *= 200 in dimensionless units), while the dotted line shows it at approximately 145 days before start of therapy (*t *= 100 in dimensionless units). From the spatial spread that occurs between these time points (82.15 dimensionless spatial units, corresponding to 0.29 cm in dimensional terms) we calculate the wave speed to approximately 0.002 cm/day. **Parameter values**: *η*_0 _= 10.0, *d*_*p *_= 0.5, *d*_*s *_= 4.8 × 10^5^, *D*_*s *_= 2.0 × 10^5^, *D*_*v *_= 1, *h*_*s *_= 1.6 × 10^6^, *s*_*β *_= 0.4, *σ*_*p *_= 3.7 × 10^4 ^with *δ *= 9 (case **A**), *δ *= 11 (case **B**) and *δ *= 11.6 (case **C**). Numerical solution obtained with Δ*x *= 25/900 for the domain size *L *= 250 with initial conditions (*p*(0, 0), *v*(0, 0), *s*(0, 0), *c*(0, 0)) = (0.01, 1, 10/12, 0) and (*p*(*x*, 0), *v*(*x*, 0), *s*(*x*, 0), *c*(*x*, 0)) = (0, 1, 10/12, 0) for *x*: 0 <*x *≤ 250.

In Figure 6 we present typical snapshots showing how the tumour cell density varies across the spatial domain when chemotherapy is first applied (at *t *= 200, in dimensionless units, in all three cases). From panel A we estimate the speed of tumour invasion to be 0.002 cm/day; this value is of the same order of magnitude as the speed of 0.009 cm/day that has been observed in gliomas [30]. We note that the spatial extents, and thus the speeds, are similar in all three cases. Linearising ahead of the wave, the *p*-equation decouples and a travelling wave analysis indicates that there is a minimum wave speed which is independent of *δ*. Calculated speeds are in close agreement with the predicted minimum speeds.

A way of measuring the therapy's effect on tumour size is to monitor the tumour's spatial extent. Here we investigate how far the invading wave front has spread by determining the length of the interval in which the tumour cell density exceeds a threshold value, *p*_*crit*_, i.e. determining *x*_*max *_so that ∀*x *∈ [*x*_*max*_, 1]: *p*(*x, t*) <*p*_*crit*_. Various imaging modalities, e.g. CT and MRI, function in this way; the tumour can only be detected wherever its density is above the "threshold of detection" (thus the real spatial extent of the tumour is in fact larger than that revealed by the medical image) [30]. In all simulations of chemotherapy we observed that therapy induces a similar response, with an initial decrease in *x*_*max*_, meaning that the therapy causes the invading wave front to recede.

**Figure 7:**
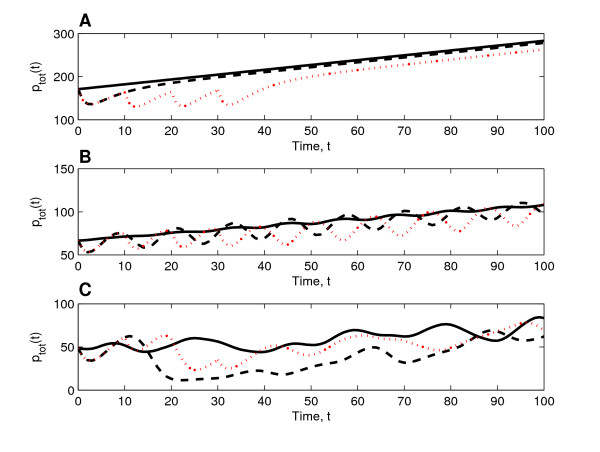
**System responses to therapy**. Three trios of curves (calculated from solutions of (11)-(15), using (27)) showing how the tumour burden, *p*_*tot*_, varies after a single bolus of chemotherapy (black dashed lines) and 4 boluses of chemotherapy (red dotted lines). Here time, *t*, represents time since therapy was initialised. **(A) **In this case, before therapy is applied, the dynamics behind the invading front evolve to a stable equilibrium. Both a single bolus and multiple boluses decrease tumour burden relative to untreated control (solid line). This persists even during the recovery phase, when the effects of the therapy wane and the tumour starts to regrow. Multiple boluses are clearly more effective than a single bolus. **(B) **When in the absence of therapy tumour invasion results in the development of regular oscillations (solid line), the therapies induce oscillations of larger amplitude during the regrowth phase. Multiple boluses are slightly more effective than a single bolus. **(C) **When irregular oscillations occur in the absence of therapy (solid line), chemotherapies lead to an increase in the amplitude of these oscillations during the recovery phase. Multiple boluses do not improve the therapeutic response. **Key**: solid lines: no therapy; black dashed lines: single bolus; red dotted lines: 4 boluses (each separated by ten time units). **Parameter values**: as in Figure 6 with *d*_*c *_= 2.0 × 10^4^, *D*_*c *_= 1.6 × 10^4^, *h*_*c *_= 1.3 × 10^3^, *k *= 0.9 and *K*_*c *_= 0.05.

In our simulations we observed that the therapy had a more pronounced effect on tumour burden than on the tumour's spatial extent. Therefore in what follows we only monitor the tumour burden, *p*_*tot*_, where *p*_*tot *_is defined by the Trapezoid rule(27)

where *p*_*i *_= *p*(*x*_*i*_, t), *x*_*i *_= *i*Δ*x *for *i *= 0, ..., *n *and Δ*x *= *L*/*n*. In Figure 7 we show how the response to both a single bolus of chemotherapy and multiple boluses depends on the underlying system dynamics. We note that the initial tumour burden, *p*_*tot*_, is highest in the case of stable tumour dynamics (Figure 7, panel A) and lowest in the case of irregular oscillations (Figure 7, panel C), whereas the initial spatial extents are similar in all three cases (see Figure 6). Since occlusion is more pronounced (*δ *being high) when the dynamics are oscillatory, the varying initial values of *p*_*tot *_reveal that stable/strong vessels promote tumour growth more effectively than unstable/weak ones. Experimental observations of ovarian carcinoma spheroids have yielded similar trends: fast-growing tumours, that increased exponentially in volume, exhibited only small variations in the density of their vasculature, which mainly consisted of mature vessels [6]. Slow-growing tumours, on the other hand, had a larger proportion of immature vessels, and exhibited larger fluctuations in both their growth rate and their vessel density [6]. In Figure 7 we observe that in all cases initially therapy decreases the tumour burden. By comparing the tumour regrowth that occurs as the drug degrades, striking differences between the different cases are revealed. When the underlying dynamics of the control are such that the system evolves to a stable equilibrium (Figure 7, panel A), the cell population increases monotonically after a single bolus of chemotherapy. In this case the effect of therapy is unambiguous: the treated tumour is always smaller than the untreated one. Furthermore, multiple injections (corresponding to one bolus every 14 days in dimensional parameters and four times as much drug being administered compared to a single dosage) are clearly more effective at controlling tumour regrowth. When the untreated tumour undergoes regular or irregular oscillations (Figure 7, panels B and C, respectively), the oscillations during the regrowth phase are of larger amplitude than for the drug-free controls, making the effect of therapy harder to evaluate and less predictable. For example, in the case of irregular oscillations multiple injections do not provide an obvious improvement compared to only one round of therapy. The increase in the amplitudes of the oscillations that the therapy induces stems from the inherent oscillatory dynamics. Specifically, the initial decrease in the tumour cell density allows the vessels to recover from occlusion throughout the spatial domain. As the therapy wanes any tumour cells that remain have an abundant supply of oxygen. Therefore, the recovering tumour cell population attains higher total cell numbers than it does when no therapy is applied. The large cell population induces more extensive vessel occlusion, causing the tumour cell population to decline again to low cell numbers. In this way the large amplitude oscillations are sustained. We remark that in panel B, where the oscillations are regular, the period of the oscillations corresponds to roughly 17 days in dimensional terms. Slower/faster oscillations can be achieved by increasing/decreasing the maximum rate of vessel proliferation, *η*_0_, and the maximum rate of vessel occlusion, *δ *(ensuring *δ*/*η*_0 _> 1). Simulations with slower/faster oscillations (and others with *D*_*v *_≠ 1) yielded similar results as those presented above.

## Discussion

In this paper we have developed a PDE model which describes tumour growth within a vascularised tissue where the tumour cells rely on the vasculature for their supply of oxygen. Analysis of a spatially homogeneous submodel, for which the oxygen profile is assumed to be quasi-steady, showed that the tumour cell and vessel densities either evolve to a stable equilibrium or undergo oscillations. We found that the latter is more likely to occur if the vessels are prone to occlusion (*δ *being large, corresponding to immature vessels) and that the oscillations arise from interactions between the tumour cells and the compliant blood vessels.

Oscillations in mathematical models of tumour growth have been described by others [5,31]. However, different mechanisms are responsible for those oscillatory dynamics. In [5] the oscillations are due to time-delays in an ODE-system, while in [31] the underlying cause is vascular adaptation induced by VEGF secretion by quiescent tumour cells. To our knowledge, this is the first model of vascularised tumour growth in which the direct interactions between the tumour cells and the vasculature lead to sustained oscillations of significant amplitude (very small oscillations were observed in the model developed by Araujo and McElwain [4]). Experimental investigations indicate that tumour cells have the capacity to occlude vessels through the pressure they exert on the vessel wall [1,2]. Furthermore, the observation that vessel occlusion is reversible (i.e. if the tumour burden is relieved by killing off tumour cells cytotoxically, then the vessel diameter increases [1,2]) leads us to believe that oscillations of the kind we report here are physically realistic and may thus contribute to spatio-temporal heterogeneities in solid tumours. At present we lack clinical data to determine whether such oscillations are clinically observable. However, our simulations (see Figure 7) suggest that oscillations could indeed be detected from spatially-averaged clinical data, provided that the data is adequately resolved in time.

Our analysis of tumour invasion due to random cell movement revealed varying spatio-temporal patterns depending on the stability of the spatially homogeneous co-existence steady state behind the invading front. Following invasion in the case of a stable equilibrium, the system settled to this steady state. In the case of an unstable equilibrium and oscillatory solutions in the spatially homogenous system, spatial effects induced either regular or irregular spatio-temporal oscillations. By approximating our system by one of *λ*-*ω *form near the Hopf bifurcation, we were able to predict the type of oscillation that will occur. This technique has been applied previously to predator-prey models where similar spatio-temporal oscillations are observed [17].

When investigating tumour response to chemotherapy we focussed on the different growth scenarios that can arise as a result of tumour invasion. We simulated chemotherapy in the form of either a single bolus or multiple, consecutive boluses; other possible modes of delivery include continuous infusion, investigated e.g. in [12]. Our numerical simulations reveal that following administration of a single bolus of doxorubicin to a tumour which, in the absence of therapy, is growing toward a steady state behind the invading front, a reduction in tumour burden is achieved. During the regrowth period that occurs while the drug is degrading, the tumour increases monotonically in size, but the size of the tumour cell population is smaller than that of the untreated control. If the tumour undergoes regular or irregular oscillations behind the invading front, the initial response is also a decrease in the tumour burden. However, during the regrowth phase, the size of the tumour cell population fluctuates strongly, more so than without therapy, and the tumour cell population attains both higher and lower values than for the drug-free cases. The therapy reinforces the underlying cause of the oscillatory dynamics (the relief of occlusion due to pressure from the expanding tumour), leading to an increase in the amplitude of the oscillations. Not only would such irregular spatio-temporal dynamics make the therapeutic effect harder to evaluate (since the timing of measurements would be crucial), but they may also explain why chemotherapy sometimes fails. In particular, our model suggests that clinical data showing short-time regression and longer-time relapse may be the result of tumours whose dynamics are oscillatory. However, in order to generate quantitative predictions, comprehensive and systematic experiments in which all model parameters can be estimated from the same cell line are urgently needed. In the absence of such data, a detailed sensitivity analysis (where system parameters are varied within physiological ranges) could provide additional information about the relative importance of individual parameters on the system dynamics.

It is interesting to compare our simulations of a single bolus of doxorubicin to the simulations presented in [15], wherein a modified version of the multiscale model developed in [31] was studied. The modifications in [15] relate specifically to the effect that tumour cells have on the pre-existing vasculature. Vessel remodelling is incorporated at each time step so that vessels surrounded by tumour cells are considered immature/destabilised, whereas those without tumour cells are considered mature. The inclusion of vessel destabilisation/maturation has pronounced effects on the therapeutic response of the system in [15]: after a single bolus, in the case where the vessels undergo destabilisation/maturation, pronounced oscillations in the tumour cell population occur as the tumour cells recover from the therapy; when destabilisation/maturation is neglected, the tumour cell recovery is more regular and the tumour cell population increases steadily. These dynamic responses are similar to the results we obtain when the tumour is exposed to a single bolus (Figure 7): during the regrowth phase, the tumour undergoes oscillations (when the underlying equilibrium is unstable) or continuously increases (when the underlying equilibrium is stable). We remark here that in our model the change from monotonically increasing tumour recovery to oscillatory tumour recovery occurs as the maximum strength of vessel occlusion is increased. Therefore, our oscillatory vessels may be regarded as more unstable than those present in tumours that recover without oscillations, a phenomenon that would be consistent with the ideas presented in [15] that vessel destabilisation introduces an irregular tumour growth pattern due to blood flow heterogeneities.

As with any mathematical description of a biological system, modifications could be made to increase the realism of our model. For example, we could investigate the effects that the angiopoietins, Ang-1 and Ang-2, have on the developing vasculature. To do this we would need to separate the vessels into two subpopulations consisting of immature vessels (destabilised by Ang-2) and mature vessels (stabilised by Ang-1). Immature vessels may be particularly sensitive to occlusion since they typically lack a pericyte covering [1]. Another obvious extension involves introducing VEGF explicitly into the model, allowing the rate of EC proliferation to be a saturating function of the VEGF concentration. We anticipate that these model extensions would yield similar qualitative behaviour, e.g. if the maximum rate of vessel occlusion (*δ*) exceeded the rate of EC proliferation (*η*_0_) then the oscillatory solutions would persist. By introducing VEGF it would also be possible to include chemotaxis with vessels moving up spatial gradients of VEGF. The inclusion of chemotaxis is likely to affect the spatio-temporal patterns that emerge as a result of tumour invasion.

Regarding the way in which we have modelled chemotherapy it is also possible to amend the model to more accurately describe doxorubicin activity. For example, we could investigate the effects of changing the functional form used to model cell kill in equation (2). As found in [14], the model predictions may depend on the numerical value of the threshold at which the rate of cell kill switches from low to high (in our model, this value is represented by the parameter *K*_*c*_). We could also investigate the tumour's response to therapy if the drug targets both tumour cells and proliferative ECs.

Despite the simplicity of the model, the simulations of doxorubicin administration presented in this paper still give considerable insight into how the response to chemotherapy may be strongly influenced by the underlying system growth dynamics. A possible direction for further research involves extending our model to distinguish between proliferating and quiescent tumour cells in order to determine how the dynamics are affected by cell heterogeneity. We could also extend our model to investigate the impact that vessel normalisation strategies, designed to increase the stability of the immature tumour vessels, have on systems with irregular spatio-temporal dynamics. Possible candidates for vascular-targetting agents include the anti-angiogenic drug DC101 which transiently increases pericyte recruitment, hence increasing vessel stability, oxygenation, and drug delivery [32]. Modelling vascular-targetting treatments could be achieved by making the occlusion parameter *δ *(which appears in equation (3)) a decreasing function of the vascular-targetting drug concentration. We anticipate that this could render the system more stable, possibly enhancing the therapy's performance.

## Conclusions

In this paper we have developed a deterministic model of vascular tumour growth formulated as a system of reaction-diffusion equations. Using a combination of numerical methods and analytical techniques we showed how the system's spatio-temporal dynamics (and, in particular, tumour-invasion patterns) are influenced by the dynamics of the associated spatially-uniform system. Depending on the interplay between the proliferating tumour cells and the collapsible vessels, we observed patterns of tumour invasion ranging from regular travelling waves to waves with highly irregular spatio-temporal structure. The latter were characterised by highly responsive, compliant vessels while the former were characterised by stiffer, less compliant vessels. Simulations of chemotherapy illustrated how treatment outcome depends on the dynamics of the untreated tumour. In particular we found that the therapeutic efficacy could be dramatically reduced by the presence of immature vessels which contribute to the emergence of oscillatory dynamics.

## Competing interests

The authors declare that they have no competing interests.

## Authors' contributions

All authors contributed to the development of the mathematical model and to the production of this manuscript. IJS performed the numerical simulations and *λ*-*ω *analysis, with particular support from MRO.

## Reviewers' comments

### Reviewer's report 1

Prof. Zvia Agur, The Institute for Medical BioMathematics (IMBM), Israel

#### Reviewer's comments

This paper focuses on vessel occlusion. Analysis of a mathematical model for vessel occlusion, in the form of a system of reaction-diffusion equations, shows that increased occlusion may give rise to sustained oscillations in tumor cell density and in vessel density. The paper suggests that oscillations in tumor size may compromise the efficacy of Doxorubicine chemotherapy. I would like to mention a few points which may be considered in order to improve the paper.

1. The basic model deliberately simplifies tumor growth and vascularisation dynamics, and couples these simple dynamics through vessel occlusion (see, e.g., Fig 2). However, occlusion is not a frequent phenomenon in tumor angiogenesis, where angiogenesis and tumor growth are always coupled through various other factors, nor is it prerequisite for oscillations in tumor and vascular dynamics (see below).

2. The paper makes some claims, which require substantiation, or should be reconciled with literature, or should be altogether eliminated. In the Discussion section (P. 15, Paragraph 3) the authors state: "To our knowledge, this is the first model of vascularised tumor growth in which the direct interactions between the tumor cells and the vasculature lead to sustained oscillations of significant amplitude". However, I believe this statement should be eliminated. Both some of the papers cited in the present manuscript and others have already showed the same result. For example, in [33], we have introduced and analysed several alternative general tumor angiogenesis models (with/without vascular maturation or with/without the effects of growth factors). Our results show that whenever a time-delay is introduced into the tumor proliferation or the neo-vascularization process, Hopf points are found, leading to oscillatory behavior. While it is recognized that time-delay will often elicit Hopf points, in [33], it was shown that the latter were to be found in any angiogenesis model with time-delay. There are other examples of angiogenesis models showing oscillatory tumor and vasculature dynamics as a result of coupling between these two processes, for example, as a result of certain ratios between growth factors' reaction rates. Suggestion: The authors should crystallize the novelty in their results and be precise about the conditions for obtaining it. In this context, I believe they should make a distinction between occlusion and hypoxia, which may be caused by many other factors.

3. Another example. On page 3 parag 2 Stamper et al write: " Analysis of the model we derive in this paper reveals that the interplay between tumor and its blood vessels may lead to strongly oscillating dynamics... if the vessels are structurally unstable". Previous models with no occlusion assumption have shown the same result. Some of them, but not all, are cited in the present manuscript (e.g., [34]).

4. One of the main conclusions of the paper is that ("Conclusions", p. 21, Parag 2): "Simulations of the chemotherapy illustrated how treatment outcome depends on the dynamics of the untreated tumor...". I believe this is already known to any scientist involved in cancer research. In any case it has been shown in many papers. I think this sentence should be suppressed.

5. I suggest that sentences such as (Page 18, parag 2) "We found that ...oscillations arise from the interactions between the tumor cells and the compliant blood vessels" would rather be eliminated.

6. I have not found any reference to the pharmakodynamics of doxorubicine. What is the assumed drug action mechanism? Why does the model represent doxorubicine and no other chemotherapy (half-life)?

Top sum up, I think that the paper will benefit from a careful editing where the main messages are spelled out consistently through the Abstract, Results, and Conclusions sections, and where the special assumptions and simplifications are recognised so that conclusions and implications are more restricted.

#### Authors' response

We agree with Professor Agur that our model is not the first one of vascular tumour growth to produce oscillatory dynamics - that is why we cite the seminal work of Agur and coworkers, amongst others. The essential novelty in our paper arises from the investigation of how the interplay between the oscillations associated with the underlying time-dependent model and diffusive effects can give rise to highly irregular spatio-temporal dynamics within the tumour mass and their impact on the tumour's response to therapy.

### Reviewer's report 2

Prof. Marek Kimmel, Department of Statistics, Rice University, USA

#### Reviewer's comments

A comment concerning the catalogue of spatial oscillatory patterns. In the paper [35] by Marciniak-Czochra and Kimmel, a new model of field carcinogenesis, is explored inspired by lungcancer precursor lesions, which includes dynamics of a spatiallydistributed population of pre-cancerous cells c(t; x), constantly supplied by an influx of mutated normal cells. Cell proliferation is controlled by growth factor molecules bound to cells, b(t; x). Free growth factor molecules g(t; x) are produced by precancerous cells and may diffuse before they become bound to other cells. The model shows an invading front of oscillatory solutions, which correspond to formation of multiple spatially isolated lesions of pre-cancerous cells and which become in the limit stable spike solutions. This effect is very different from the one obtained in Stamper et al. (although some analogies between oxygen and growth factor supply exist), among other because only one of the three equations features a diffusion term.

#### Authors' response

We thank Professor Kimmel for directing us to this interesting publication. There are some similarities with our work, in that we consider diffusion-driven instability (DDI) as a mechanism for destabilising spatially homogeneous solutions. However an important difference between our work and that of Marciniak-Czochra and Kimmel is that we also use lambda-omega analysis to investigate the stability of time-periodic model solutions to spatial perturbations. In this way we provide a possible explanation for the highly irregular spatio-temporal dynamics that characterise many vascularised tumours and show that such dynamics may have a deleterious impact on the tumour's response to chemotherapy.

## Supplementary Material

Additional file 1 **Appendix**. In the pdf file Appendix.pdf we discuss our model parameters and estimate values for some of them. We also give additional information relating to the *λ*-*ω *analysis of our model. Click here for file
