# Supplementary material for: Oscillatory dynamics in a model of vascular tumour growth - implications for chemotherapy
Source: Biol Direct. 2010 Apr 20;5:27. doi: 10.1186/1745-6150-5-27 (PMC2877015; doi:10.1186/1745-6150-5-27)
Supplement: Additional file 1 — Appendix. In the pdf file Appendix.pdf we discuss our model parameters and estimate values for some of them. We also give additional information relating to the λ-ω analysis of our model. [file 1745-6150-5-27-S1.PDF]

Appendix
Oscillatory dynamics in a model of vascular tumour growth – implications for chemotherapy

I. J. Stamper, M. R. Owen, P. K. Maini, H. M. Byrne

In section A below we discuss and estimate realistic values for some of our model parameters. In section B we give additional information relating to the λ-ω analysis of our model.

A Parameter values

The model variables and the dimensional parameters are summarised in Tables 1 and 2, respectively. Below we discuss how we have estimated the dimensional model parameters and their dimensionless equivalents. We summarise our estimates of the dimensionless parameters and parameter groupings in Table 3. We remark that the parameter values employed are illustrative only since they are obtained from different tumours and diverse experiments.

**Variable**	**Dimension**	**Description**
c_b	ML^{-3}	Concentration of drug in the blood<br>(mass per unit plasma volume)
c	ML^{-3}	Concentration of drug in the tumour<br>(mass per unit tissue volume)
p	L^{-3}	Density of proliferating tumour cells<br>(number of cells per unit tissue volume)
s	1	Concentration of oxygen<br>(oxygen volume per unit tissue volume)
v	L^{-1}	Density of vasculature<br>(vessel surface area per unit tissue volume)

Table 1: Model variables

A.1 Estimation of β, d_p and d_p^*

An often-quoted parameter is the potential doubling time, T_{pot}, this being defined as the time taken for a tumour to double in size if there were no cell loss (in an *in vitro* situation this may be very close to the real doubling time) [36]. In [37] T_{pot} was estimated to be 22 hours for carcinoma cells. If we fix $T_{pot} = 1$ day, then

Parameter	Dimension	Description	Value [Ref.]/(Sect.)
β	T^{-1}	Maximum tumour growth rate	0.69/day (A.1)
c_0	ML^{-3}	Maximum drug concentration in the blood	
d_c	T^{-1}	Decay rate of drug in tissue	$1.38 * 10^4$/day (A.7)
d_p	T^{-1}	Apoptosis rate of tumour cells	
d_s	T^{-1}	Rate of oxygen consumption by other cells	
D_c	L^2T^{-1}	Diffusion coefficient of drug	1.6×10^{-6} cm^2/s [12]
D_p	L^2T^{-1}	Diffusion coefficient of tumour cells	10^{-10} cm^2/s (A.2)
D_s	L^2T^{-1}	Diffusion coefficient of oxygen	2×10^{-5} cm^2/s [40]
D_v	L^2T^{-1}	Diffusion coefficient of vessels	10^{-10} cm^2/s [41]
δ	T^{-1}	Maximum rate of vessel occlusion	
η_0	T^{-1}	Rate of vessel growth	0.2-0.56/h [42]
h_c	LT^{-1}	Vascular permeability of drug	1.0×10^{-4} cm/s [12]
h_s	LT^{-1}	Vascular permeability of oxygen	0.001-0.13 cm/s [47]
k	T^{-1}	Decay rate of drug in the blood	0.64 (A.7)
K_c	ML^{-3}	Drug concentration at which rate of tumour cell kill is half-maximal	
K_δ	L^{-2}	Value of p/v where rate of vessel occlusion attains half-maximal value	4.5×10^5 cells/mm^2 (A.5)
s_b	1	Oxygen concentration in the blood	
s_β	1	Oxygen concentration at which tumour cell proliferation attains half-maximal value and below which cells tend to become quiescent	
σ_p	L^3T^{-1}	Rate of oxygen consumption by tumour cells	
$T_{1/2}$	T	Half-life of doxorubicin	26 h [15]
T_{pot}	T	Potential doubling time of carcinoma cells	22 h [37]
V_0	L^{-1}	Vessel carrying capacity	10/mm (A.4)

Table 2: Model parameters and parameter values from references, or estimates from appendix sections

Parameter	Estimated value
d_c^*	2.0×10^4
D_c^*	1.6×10^4
D_s^*	2×10^5
D_v^*	1
η_0^*	$7 - 20$
h_c^*	1.3×10^3
h_s^*	$1.3 \times 10^4 - 1.6 \times 10^6$
k^*	0.9

Table 3: Estimates of dimensionless parameters (for values used in simulations, see each figure legend)

we estimate β, the maximum rate of proliferation, to be $\beta = \ln 2/T_{pot} \approx 0.69$/day. Once β has been fixed, it follows that the maximum growth rate, λ_{max}, where $\lambda_{max} = \beta - d_p$, depends on the rate of apoptosis, d_p. In practice the rates of proliferation and apoptosis may vary considerably and hence so may $d_p^* = d_p/\beta$ (however, assuming net growth, $0 < d_p^* < 1$ always holds). Throughout the paper we use different values of d_p^* ranging from 0.5 to 0.8, as specified in each figure legend.

A.2 Estimation of D_p, D_v, D_s, D_v^* and D_s^*

Estimates of D_p, the random motility coefficient of tumour cells, vary from $D_p \approx 10^{-9}$ cm^2/s [38] to $D_p \in [6.9 \times 10^{-11}, 3.5 \times 10^{-10}]$ cm^2/s [39]. Following [40] and [41] we fix the diffusion coefficient of oxygen and the random motility coefficient of ECs so that $D_s = 2 \times 10^{-5}$ cm^2/s and $D_v = 10^{-10}$ cm^2/s. By taking $D_p = 10^{-10}$ cm^2/s the above estimates yield $D_v^* = 1$ and $D_s^* = 2 \times 10^5$.

A.3 Estimation of η_0 and η_0^*

In [42] the maximum rate of EC proliferation was estimated to be in the range of 0.2-0.56/h. This corresponds to a range of 4.8-13.44/day for our parameter η_0. Thus, with $\beta = 0.69$/day as given above, $\eta_0^* = \eta_0/\beta$ is in the range 7-20. In our simulations of chemotherapy we use the value $\eta_0^* = 10$.

A.4 Estimation of V_0

The background level of vascularisation (vascular surface area per unit tissue volume) for normal tissues, V_0, is tissue-specific and has been estimated to be 13/mm in skeletal muscle [43], 11.5/mm in cortical parenchyma [44] and 7.63/mm in medullary tissue [44]. Therefore we take $V_0 = 10$/mm. In renal tumour tissue higher and lower values have been reported for different tumours, ranging from 3.5/mm to 12.1/mm [44]. Other estimates of V_0 for tumours lie within the range 3.6/mm to 27/mm [45, 24].

A.5 Estimation of K_δ

The cell density of normal colon tissue is approximately 2300 cells/mm^2 [46]. When the compression of tumour vessels was investigated in [1], open vessels were found to be surrounded by tumour cells at a density of 3700 cells/mm^2, while the cell density around vessels with closed lumen was 5700 cells/mm^2. We assume that the rate of occlusion is half-maximal at a cell density of 4500 cells/mm^2, and a vascular surface area per unit volume of 10/mm. To obtain K_δ we convert the cell density to cells per unit volume by assuming that the

tumour cells have a diameter of 10 μm (normally it is in the range between 10 and 100 μm [38]) and that all the cells lie in the same plane so that 4500 cells occupy a volume of 1mm^2 $\times$ 10 μm, giving a cell density of 4.5×10^6 cells/mm^3. In this way we estimate $K_\delta = 4.5 \times 10^5$ cells/mm^2.

A.6 Estimation of h_s and h_s^*

In [47] values of the transcapillary permeability of oxygen between 0.001 and 0.13 cm/s were given based on various estimates. Using $V_0 = 10$/mm and $\beta = 0.69$/day, we deduce that $h_s^* = \frac{h_s V_0}{\beta} \approx 1.3 \times 10^4$ with $h_s = 0.001$ and $h_s^* = \frac{h_s V_0}{\beta} \approx 1.6 \times 10^6$ with $h_s = 0.13$. In the simulations we present in this paper we use the latter value.

A.7 Estimation of D_c, h_c, k and d_c and their dimensionless equivalents

We assume that the cytotoxic drug of choice is doxorubicin and derive parameters corresponding to this drug. From [12] we obtain the value of the diffusion coefficient of doxorubicin to be 1.6×10^{-6} cm^2/s, which, with the value of D_p as above, gives $D_c^* = 1.6 \times 10^4$.

The vascular permeability of doxorubicin is given in [12] as 1.0×10^{-4} cm/s. With our values of β and V_0 as above we obtain that $h_c^* = \frac{h_c V_0}{\beta} \approx 1.3 \times 10^3$.

The half-life of doxorubicin in plasma is $T_{1/2} = 26$ h [15]. Therefore, with $k = \frac{-\ln(0.5)}{T_{1/2}}$, we obtain $k^* = \frac{k}{\beta} \approx 0.9$.

To obtain an estimate for d_c, the rate of drug uptake in the tumour tissue, we utilise the model in [12] where the uptake of drug in the extracellular space of the tumour was accounted for by the term $d_c V_{max}(\frac{c_e}{c_e + K_e \phi} - \frac{c_i}{c_i + K_i})$, where c_e and c_i denote the extracellular and intracellular drug concentration, respectively, and $d_c = 6 \times 10^8$cells/ml, $V_{max} = 0.28 \times 10^{-14}$g/cells/min, $K_e = 0.219 \times 10^{-6}$g/ml and $\phi = 0.4$ are positive constants. We take our decay constant, d_c, to be half the maximum value in [12], i.e. $d_c = \frac{d_c V_{max}}{2 K_e \phi}$ and, with the estimates given above, we therefore have that $d_c \approx 1.38 * 10^4$/day and $d_c^* = \frac{d_c}{\beta} \approx 2.0 \times 10^4$.

B λ-ω analysis

A λ-ω system is one of the form [27]:

$$\frac{\partial p}{\partial t} = \frac{\partial^2 p}{\partial x^2} + \lambda_0 p - \omega_0 v - (\lambda_1 p + \omega_1 v)(p^2 + v^2), \quad \text{(A-1)}$$

$$\frac{\partial v}{\partial t} = \frac{\partial^2 v}{\partial x^2} + \omega_0 p + \lambda_0 v + (\omega_1 p - \lambda_1 v)(p^2 + v^2). \quad \text{(A-2)}$$

The spatially homogeneous version of this system has an unstable steady state at $(p, v) = (0, 0)$ with a stable circular limit cycle of radius $\sqrt{\lambda_0/\lambda_1}$ [27]. The full system (A-1)-(A-2) possesses a one-parameter family of periodic wave train solutions [48, 27]:

$$p = Rcos[\omega(R)t \pm \lambda(R)^{1/2}x], \quad v = Rsin[\omega(R)t \pm \lambda(R)^{1/2}x], \tag{A-3}$$

where R is the wave amplitude and $\lambda(r)$, $\omega(r)$ and r are given by

$$\lambda(r) = \lambda_0 - \lambda_1 r^2, \quad \omega(r) = \omega_0 + \omega_1 r^2 \quad \text{and} \quad r = (p^2 + v^2)^{1/2}. \tag{A-4}$$

Furthermore, it is possible to show that the solutions (A-3) are linearly stable to perturbations if the following criterion is satisfied [48, 27]:

$$4(\lambda(R)) \left[1 + \left(\frac{\omega'(R)}{\lambda'(R)}\right)^2\right] + R\lambda'(R) \leq 0. \tag{A-5}$$

In [27] Sherratt studied the solutions of (A-1)-(A-2) that evolve from initial conditions that decay exponentially in space and correspond to the rear of the invading front. By transforming to polar co-ordinates and employing a combination of analytical and numerical techniques, Sherratt demonstrated that the amplitude of the periodic wave train that develops behind the invasive front is given by

$$R = \left[\frac{2\lambda_0}{\omega_1^2}\left(\sqrt{\lambda_1^2 + \omega_1^2} - \lambda_1\right)\right]^{1/2}. \tag{A-6}$$

By substituting (A-6) into (A-5) the criterion

$$\frac{\omega_1^2}{\lambda_1^2} < 1.148 \tag{A-7}$$

for wave stability can be obtained.

Before we can apply these results to our model it is necessary first to approximate it by one of the form (A-1)-(A-2) in the neighbourhood of the Hopf bifurcation and hence identify values of ω_1^2 and λ_1^2 for our system. In order to do this we need to identify a parameter that gives rise to a Hopf bifurcation as it is varied. Therefore we investigate the spatially homogeneous version of equations (26)-(27) (with $\sigma_p = 0$ for simplicity). We note that for the corresponding spatially homogeneous system, the nullclines and phase planes are similar to those of the system with $\sigma_p \neq 0$ (cases (a) and (b) in Figure 1), with the exception that the p-nullcline (previously given by (23)) is now a straight line on which $v = d_p s_\beta d_s/(1 - d_p s_\beta - d_p)$. This means that the steady state

solutions, (p_{ss}, v_{ss}), are given by

$$v = \frac{d_p s_\beta d_s}{1 - d_p s_\beta - d_p} \equiv v_{ss}, \quad p = \frac{v_{ss}(v_{ss} - 1)}{1 - \delta/\eta_0 - v_{ss}} \equiv p_{ss}. \tag{A-8}$$

If we assume that $\delta/\eta_0 > 1$ and hence that oscillations can occur, then a relevant bifurcation parameter is d_s. As before, increasing d_s corresponds to moving the p-nullcline to the right and when d_s exceeds a certain critical value a Hopf bifurcation occurs (see Figure 1, panel B). This critical value occurs when $trA = 0$, where $A = (a_{ij})$ is the Jacobian matrix. By setting

$$trA|_{(p,v)=(p_{ss},v_{ss})} = 0 \tag{A-9}$$

and then solving for d_s we obtain

$$d_s = \frac{-(1 - d_p s_\beta - d_p)[(\delta - \eta_0) \pm \sqrt{\delta(\delta - \eta_0)}]}{\eta_0 s_\beta d_p}. \tag{A-10}$$

By imposing $\eta_0 - \delta < 0 < 1 - d_p s_\beta - d_p$ for oscillatory solutions and a non-negative co-existence steady state, we conclude that

$$d_s = \frac{-(1 - d_p s_\beta - d_p)[(\delta - \eta_0) - \sqrt{\delta(\delta - \eta_0)}]}{\eta_0 s_\beta d_p} \equiv d_s^{crit}, \tag{A-11}$$

is the relevant positive solution of (A-10).

Following [17], for a system like (26)-(27) (with $D_v = 1$), in the λ-ω-form (also referred to as the "normal form" [28, 17]), λ_0 is the leading order term in a regular Taylor series expansion of the real part of the (complex conjugate) pair of eigenvalues of the Jacobian matrix, i.e.

$$\lambda_0 = (d_s - d_s^{crit})\frac{\partial(Re(\gamma))}{\partial d_s}\Bigg|_{d_s=d_s^{crit}} = (d_s - d_s^{crit})\frac{\partial}{\partial d_s}\left(\frac{a_{11} + a_{22}}{2}\right)\Bigg|_{d_s=d_s^{crit}}. \tag{A-12}$$

In this expansion the small parameter is $(d_s - d_s^{crit})$, and we restrict attention to values of d_s which are close to d_s^{crit}. In (A-12) γ denotes the complex conjugate pair of eigenvalues of $A = (a_{ij})$, the Jacobian matrix, at $(p, v) = (p_{ss}, v_{ss})$. Additionally, ω_0 is the imaginary part of the eigenvalues of A, again evaluated at the steady state values at which the Hopf bifurcation occurs, so that:

$$\omega_0 = Im(\gamma)|_{d_s=d_s^{crit}} = \sqrt{a_{12}|a_{21}|}\Bigg|_{d_s=d_s^{crit}}. \tag{A-13}$$

To determine λ_1 and ω_1, we set $d_s \equiv d_s^{crit}$ since corrections due to variation in d_s only affect higher order terms in the normal form [17]. Following [17] we make a linear change of variables

$$\hat{p} = \frac{p - p_{ss}}{\alpha}, \quad \hat{v} = \frac{v - v_{ss}}{\beta}, \tag{A-14}$$

which allow the linear part of (26)-(27) to be in normal form. In what follows we choose α and β so that (26)-(27) (with $\sigma_p = 0$ and $D_v = 1$) transform to give

$$\frac{\partial \hat{p}}{\partial t} = \frac{\partial^2 \hat{p}}{\partial x^2} - \omega_0 \hat{v} + \bar{P}(\hat{p}, \hat{v}) \tag{A-15}$$

and

$$\frac{\partial \hat{v}}{\partial t} = \frac{\partial^2 \hat{v}}{\partial x^2} + \omega_0 \hat{p} + \bar{V}(\hat{p}, \hat{v}), \tag{A-16}$$

where $\bar{P}$ and $\bar{V}$ satisfy

$$\bar{P}(0,0) = \frac{\partial \bar{P}(0,0)}{\partial \hat{v}} = \frac{\partial \bar{P}(0,0)}{\partial \hat{p}} = 0, \tag{A-17}$$

$$\bar{V}(0,0) = \frac{\partial \bar{V}(0,0)}{\partial \hat{v}} = \frac{\partial \bar{V}(0,0)}{\partial \hat{p}} = 0. \tag{A-18}$$

Substituting (A-14) into (26)-(27), we obtain

$$\frac{\partial \hat{p}}{\partial t} = \frac{\partial^2 \hat{p}}{\partial x^2} + \frac{1}{\alpha} P(\hat{p}, \hat{v}) \equiv \frac{\partial^2 \hat{p}}{\partial x^2} + P^*(\hat{p}, \hat{v}) = \frac{\partial^2 \hat{p}}{\partial x^2} - \omega_0 \hat{v} + P^*(\hat{p}, \hat{v}) + \omega_0 \hat{v} \tag{A-19}$$

and

$$\frac{\partial \hat{v}}{\partial t} = \frac{\partial^2 \hat{v}}{\partial x^2} + \frac{1}{\beta} V(\hat{p}, \hat{v}) \equiv \frac{\partial^2 \hat{v}}{\partial x^2} + V^*(\hat{p}, \hat{v}) = \frac{\partial^2 \hat{v}}{\partial x^2} + \omega_0 \hat{p} + V^*(\hat{p}, \hat{v}) - \omega_0 \hat{p}. \tag{A-20}$$

Since $trA|_{(p,v,d_s)=(p_{ss},v_{ss},d_s^{crit})} = 0$ and $a_{11}|_{(p,v)=(p_{ss},v_{ss})} = 0$, as follows from (26), we deduce that $a_{11} = a_{22} = 0$, and, therefore,

$$\frac{\partial P^*}{\partial \hat{p}} = \frac{1}{\alpha} \frac{\partial P}{\partial p} \frac{dp}{d\hat{p}} = \frac{1}{\alpha} a_{11} \frac{dp}{d\hat{p}} = 0 \tag{A-21}$$

and

$$\frac{\partial V^*}{\partial \hat{v}} = \frac{1}{\beta} \frac{\partial V}{\partial v} \frac{dv}{d\hat{v}} = \frac{1}{\beta} a_{22} \frac{dv}{d\hat{v}} = 0. \tag{A-22}$$

By now requiring

$$\frac{\partial P^*}{\partial \hat{v}} = \frac{1}{\alpha} \frac{\partial P}{\partial v} \frac{dv}{d\hat{v}} = \frac{1}{\alpha} \frac{\partial P}{\partial v} \beta = \frac{1}{\alpha} a_{12} \beta = -\omega_0 \tag{A-23}$$

and

$$\frac{\partial V^*}{\partial \hat{p}} = \frac{1}{\beta} \frac{\partial V}{\partial p} \frac{dp}{d\hat{p}} = \frac{1}{\beta} \frac{\partial V}{\partial p} \alpha = \frac{1}{\beta} a_{21} \alpha = \omega_0 \tag{A-24}$$

it follows that

$$\bar{P}(\hat{p}, \hat{v}) \equiv P^*(\hat{p}, \hat{v}) + \omega_0 \hat{v} \tag{A-25}$$

and

$$\bar{V}(\hat{p},\hat{v}) \equiv V^*(\hat{p},\hat{v}) - \omega_0 \hat{p} \tag{A-26}$$

satisfy (A-17)-(A-18) as required. Thus we choose

$$\beta = 1 \tag{A-27}$$

and

$$\alpha = -a_{12}/\omega_0 \tag{A-28}$$

so that (A-23)-(A-24) are satisfied. (We note from (A-13) that $\alpha = -a_{12}/\omega_0 = -\sqrt{a_{12}}/\sqrt{|a_{21}|} = \omega_0/a_{21}$.)

Once in the form (A-15)-(A-16), λ_1 and ω_1 (which, together with λ_0 and ω_0 in (A-12)-(A-13), complete the normal form) are given by the formulae [17]:

$$\begin{aligned}\lambda_1 = &-\frac{1}{16}[\bar{P}_{\hat{p}\hat{p}\hat{p}} + \bar{P}_{\hat{p}\hat{v}\hat{v}} + \bar{V}_{\hat{p}\hat{p}\hat{v}} + \bar{V}_{\hat{v}\hat{v}\hat{v}}] \\ &+ \frac{1}{16\omega_0}[\bar{P}_{\hat{p}\hat{p}}\bar{V}_{\hat{p}\hat{p}} - \bar{P}_{\hat{v}\hat{v}}\bar{V}_{\hat{v}\hat{v}} - \bar{P}_{\hat{p}\hat{v}}(\bar{P}_{\hat{p}\hat{p}} + \bar{P}_{\hat{v}\hat{v}}) + \bar{V}_{\hat{p}\hat{v}}(\bar{V}_{\hat{v}\hat{v}} + \bar{V}_{\hat{p}\hat{p}})]\end{aligned} \tag{A-29}$$

and

$$\begin{aligned}\omega_1 = &\frac{1}{16}[\bar{V}_{\hat{p}\hat{p}\hat{p}} + \bar{V}_{\hat{p}\hat{v}\hat{v}} - \bar{P}_{\hat{p}\hat{p}\hat{v}} - \bar{P}_{\hat{v}\hat{v}\hat{v}}] + \frac{1}{48\omega_0}[\bar{P}_{\hat{p}\hat{p}}(\bar{V}_{\hat{p}\hat{v}} - \bar{P}_{\hat{v}\hat{v}}) + \bar{V}_{\hat{v}\hat{v}}(\bar{P}_{\hat{v}\hat{v}} - \bar{V}_{\hat{p}\hat{p}}) \\ &- 3\bar{P}_{\hat{v}\hat{v}}(\bar{V}_{\hat{p}\hat{v}} + \bar{P}_{\hat{v}\hat{v}}) - 3\bar{V}_{\hat{p}\hat{p}}(\bar{P}_{\hat{p}\hat{v}} + \bar{V}_{\hat{p}\hat{p}}) - 2(\bar{P}_{\hat{p}\hat{p}} + \bar{P}_{\hat{v}\hat{v}} - \bar{V}_{\hat{p}\hat{v}})^2 - 2(\bar{V}_{\hat{p}\hat{p}} + \bar{V}_{\hat{v}\hat{v}} - \bar{P}_{\hat{p}\hat{v}})^2],\end{aligned} \tag{A-30}$$

where the subscripts denote partial derivatives [17], e.g. $\bar{P}_{\hat{p}\hat{v}\hat{v}} \equiv \partial^3\bar{P}/\partial\hat{p}\partial\hat{v}^2$, all partial derivatives being evaluated at $\hat{p} = \hat{v} = 0$.

After determining $\bar{P}$ and $\bar{V}$ and using (A-29)-(A-30) to calculate λ_1 and ω_1, the stability of the periodic waves behind the invading front can be determined from (A-7). We remark that, for fixed values of η_0 and s_β, λ_1 and ω_1, and thus the stability of the waves (see (A-7) and (A-29)-(A-30)), depend solely on δ and d_p (see Figure 3). However, before solving (26)-(27) for a case where the parameter values are in the stable region (Figure 4) and a case of instability (Figure 5), we must use (A-11) in each case to determine the corresponding value of d_s^{crit}, since the predicted stability only holds for values of d_s near d_s^{crit} [27]. In Figure 3 we have marked the contours where d_s^{crit} is constant by solid lines with their respective values. Note that in Figure 3 we require $d_p < 1/(1+s_\beta)$ for the co-existence steady state to be positive and $\delta > \eta_0$ for it to be unstable. Thus by varying only δ, the maximum rate of vessel occlusion, it is possible to obtain a parameter region corresponding to a stable equilibrium behind the invading front (for $\delta < \eta_0 = 0.5$) in addition to the regions of regular and irregular oscillations (depicted in Figure 3).

References

[1] Padera TP, Stoll BR, Tooredman JB, Capen D, di Tomaso E, Jain RK: **Cancer cells compress intratumour vessels** *Nature* 2004, **427**:695.

[2] Griffon-Etienne G, Boucher Y, Brekken C, Suit HD, Jain RK: **Taxane-induced apoptosis decompresses blood vessels and lowers interstitial fluid pressure in solid tumours: clinical implications**. *Cancer Res* 1999, **59**:3776-3782.

[3] Breward CJW, Byrne HM, Lewis CE: **A multiphase model describing vascular tumour growth**. *Bull Math Biol* 2003, **65**:609-640.

[4] Araujo RP, McElwain DLS: **The role of mechanical host-tumour interactions in the collapse of tumour blood vessels and tumour growth dynamics**. *J Theor Biol* 2006, **238**:817-827.

[5] Arakelyan L, Vainstein V, Agur Z: **A computer algorithm describing the process of vessel formation and maturation, and its use for predicting the effects of anti-angiogenic and anti-maturation therapy on vascular tumour growth**. **Angiogenesis** 2002, **5**:203-214.

[6] Arakelyan L, Merbl Y, Agur Z: **Vessel maturation effects on tumour growth: validation of a computer model in implanted human ovarian carcinoma spheroids**. *Eur J Canc* 2005, **41**:159-167.

[7] Matzavinos A, Chaplain MAJ: **Travelling-wave analysis of a model of the immune response to cancer**. *C. R. Biologies* 2004, **327**:995-1008.

[8] Matzavinos A, Chaplain MAJ: **Mathematical modelling of the spatio-temporal response of cytotoxic T-lymphocytes to a solid tumour**. *Math Med Biol* 2004, **21**:1-34.

[9] Owen MR, Sherratt JA: **Pattern formation and spatiotemporal irregularity in a model for macrophage-tumour interactions**. *J Theor Biol* 1997, **189**:63-80.

[10] Jain RK: **Determinants of tumor blood flow: a review**. *Canc Res* 1988, **48**:2641-2658.

[11] Jain RK: **Normalization of tumor vasculature: an emerging concept in antiangiogenic therapy**. *Science* 2005, **307**:58-62.

[12] El-Kareh AW, Secomb TW: **A mathematical model for comparison of bolus injection, continuous infusion, and liposomal delivery of doxorubicin to tumor cells**. *Neoplasia* 2000, **2**:325-338.

[13] Rahman A, Carmichael D, Harris M, Roh JK: **Comparative pharmacokinetics of free doxorubicin and doxorubicin entrapped in cardiolipin liposomes**. *Canc Res* 1986, **46**:2295-2299.

[14] Jackson TL: **Intracellular accumulation and mechanism of action of doxorubicin in a spatio-temporal tumor model**. *J Ther Biol* 2003, **220**:201-213.

[15] Ribba B, Marron K, Agur Z, Alarcón T, Maini PK: **A mathematical model of doxorubicin treatment efficacy for non-Hodgkin's lymphoma: investigation of the current protocol through theoretical modelling results**. *Bull Math Biol* 2005, **67**:79-99.

[16] Sherratt JA, Eagan BT, Lewis MA: **Oscillations and chaos behind predator-prey invasion: mathematical artifact or ecological reality?**. *Phil Trans R Soc Lond B* 1997, **352**:21-38.

[17] Sherratt JA: **Periodic travelling waves in cyclic predator-prey systems**. *Ecology Letters* 2001, **4**:30-37.

[18] Orme ME, Chaplain MAJ: **A mathematical model of vascualar tumour growth and invasion**. *Math. Comput. Modelling* 1996, **23**:43-60.

[19] Secomb TW, Hsu R, Dewhirst MW: **Synergistic effects of hyperoxic gas breathing and reduced oxygen consumption on tumor oxygenation: a thereoretical model**. *Int J Radiation Oncology Biol Phys* 2004, **59**:572-578.

[20] Edelstein-Keshet L: *Mathematical models in biology*, McGraw-Hill Inc, 1988.

[21] Casciari JJ, Sotirchos SV, Sutherland RM: **Variations in tumour cell growth rates and metabolism with oxygen concentration, glucose concentration and extracellular pH**. *J Cell Physiol* 1992, **151**:386-394.

[22] Fornari FA, Randolph JK, Yalowich JC, Ritke MK, Gewirtz DA, *Interference by doxorubicin with DNA unwinding in MCF-7 breast tumor cells. Mol Pharm* 1994, **45**:649-656.

[23] Webb SD, Owen MR, Byrne HM, Murdoch C, Lewis CE: **Macrophage-based anti-cancer therapy: modelling different modes of tumour targeting**. *Bull Math Biol* 2007, **69**:1747-1776.

[24] Jain RK: **Transport of molecules, particles, and cells in solid tumors**. *Annu Rev Biomed Eng* 1999, **01**:241-263.

[25] **NAG Fortran library routine document D03PCF/D03PCA** [http://www.nag.co.uk/numeric/fl/manual20/pdf/D03/d03pcf.pdf]

[26] Murray JD: *Mathematical Biology*, Springer Verlag, 2nd edition, 1993.

[27] Sherratt JA: **Invading wave fronts and their oscillatory wakes are linked by a modulated travelling phase resetting wave**. *Physica D* 1998, **117**:145-166.

[28] Guckenheimer J, Holmes P: *Nonlinear oscillations, dynamical systems, and bifurcations of vector fields*, Volume 42, Springer-Verlag, 1983.

[29] Malchow H, Petrovskii SV: **Dynamical stabilization of an unstable equiibrium in chemical and biological systems**. *Math Comp Mod* 2002, **36**:307-319.

[30] Swanson KR, Bridge C, Murray JD, Alvord Jr EC: **Virtual and real brain tumors: using mathematical modeling to quantify glioma growth and invasion**. *J Neurological Sci* 2003, **216**:1-10.

[31] Byrne HM, Owen MR, Alarcón T, Murphy J, Maini PK: **Modelling the response of vascular tumours to chemotherapy: a multiscale approach**. *Math Mod Meth Appl Sci* 2006, **16**(Suppl 1):1219-1241.

[32] Lin MI, Sessa WC: **Antiangiogenic therapy: creating a unique "window" of opportunity**. *Cancer Cell* 2004, **6**:529-531.

[33] Agur Z, Arakelyan L, Daugulis P, Ginosar Y: **Hopf point analysis for angiogenesis models**. *Discrete and Continuous Dynamical Systems Series B* 2004, **4**:29-38.

[34] Arakelyan L, Merbl Y, Daugulis P, Ginosar Y, Vainstein V, Selitser V, Kogan Y, Harpak H, Agur Z: **Multiscale analysis of angiogenic dynamics and therapy**. In *Cancer Modelling and Simulation.* Edited by Preziosi L. Chapman and Hall/CRC; 2003:185-220.

[35] Marciniak-Czochra A, Kimmel M: **Reaction-difusion model of early carcinogenesis: The effects of influx of mutated cells**. *Math. Model. Nat. Phenom.* 2008, **3**:90-114.

[36] Shibui S, Hoshino T, Vanderlaan M, Gray JW: **Double labeling with iodo- and bromodeoxyuridine for cell kinetics studies**. *The Journal of Histochemistry and Cytochemistry* 1989, **37**:1007-1011.

[37] Tannock IF: **Population kinetics of carcinoma cells, capillary endothelial cells, and fibroblasts in a transplanted mouse mammary tumor**. *Canc Res* 1970, **30**:2470-2476.

[38] Anderson ARA: **A hybrid mathematical model of solid tumour invasion: the importance of cell adhesion**, *Math Med Biol* 2005, **22**:163-186.

[39] Levine HA, Pamuk S, Sleeman BD, Nilsen-Hamilton M: **Mathematical modeling of capillary formation and development in tumor angiogenesis: penetration into the stroma**. *Bull Math Biol* 2001, **63**:801-863.

[40] Grote J, Süsskind R, Vaupel P: **Oxygen diffusivity in tumor tissue (DS-Carcinosarcoma) under temperature conditions within the range of 20-40C**. *Eur J Phys* 1977, **372**:37-42.

[41] Chaplain MAJ: **Mathematical modelling of angiogenesis**. *J Neuro-Oncology* 2000, **50**:37-51.

[42] Chaplain MAJ, Stuart AM: **A model mechanism for the chemotactic response of endothelial cells to tumour angiogenesis factor**. *IMA J Math Appl Med Biol* 1993, **10**:149-168.

[43] Brix G, Bahner ML, Hoffmann U, Horvath A, Schreiber W: **Regional blood flow, capillary permeability, and compartmental volumes: measurement with dynamic CT – initial experience**. *Radiology* 1999, **210**:269-276.

[44] Köhler HH, Barth PJ, Siebel A, Gerharz E, Bittinger A: **Quantitative assessment of vascular surface density in renal cell carcinomas**. *Br J Urology* 1996, **77**:650-654.

[45] Grunstein J, Roberts WG, Mathieu-Costello O, Hanahan D, Johnson RS, **Tumor-derived expression of vascular endothelial growth factor is a critical factor in tumor expansion and vascular function**. *Canc Res* 1999, **59**:1592-1598.

[46] Tsuji T, Sasaki Y, Tanaka M, Hanabata N, Hada R, Munakata A: **Microvessel morphology and vascular endothelial growth factor expression in human colonic carcinoma with or without metastasis**. *Lab Invest* 2002, **82**:555-562.

[47] Zhang W, Edwards A: **Oxygen transport across vasa recta in the renal medulla**. *Am J Physiol Heart Circ Physiol* 2002, **283**:H1042-H1055.

[48] Kopell N, Howard LN: **Plane wave solutions to reaction-diffusion equations**. *Stud Appl Math* 1973, **52**:291-328.
